# Eye-movement efficiency and sight-reading expertise in woodwind players

**DOI:** 10.16910/jemr.12.2.6

**Published:** 2019-08-31

**Authors:** Katie Zhukov, Sieu Khuu, Gary E. McPherson

**Affiliations:** University of Queensland, Australia; University of New South Wales, Australia; University of Melbourne, Australia

**Keywords:** eye movement, sight-reading, fixation duration, fixation, expertise, task difficulty

## Abstract

The ability to sight-read traditional staff notation is an important skill for all classically trained musicians. Up until now, however, most research has focused on pianists, by comparing experts and novices. Eye movement studies are a niche area of sight-reading research, focusing on eye-hand span and perceptual span of musicians, mostly pianists. Research into eye movement of non-piano sight-reading is limited. Studies into eye movement of woodwind sight-reading were conducted in the 1980s and early 2000s, highlighting the need for new research using modern equipment. This pilot study examined the eye movements of six woodwind (flute, clarinet) undergraduates of intermediate-to-advanced skill level during sight-reading of scores of increased difficulty. The data was analysed in relation to expertise level and task difficulty, focusing on numbers of fixations and fixation durations. The results show that as music examples became more difficult the numbers of fixations increased and fixation durations decreased; more experienced players with better sight-reading skills required less time to process musical notation; and participants with better sightreading skills utilised fewer fixations to acquire information visually. The findings confirm that the efficiency of eye movements is related to instrumental and sightreading expertise, and that task difficulty affects eye movement strategies.

## Introduction

Expert sight-reading (SR) – the ability to read new music fluently at first sight or “prima vista” – is an important skill for pianists, particularly those working as accompanists, repetiteurs and orchestral pianists (1). Pianists in such careers are required to play complex scores with minimal or no preparation and therefore, have to rely on their ability to process musical notation and decode this information into a competent performance immediately (2). Expert SR is a process in which “expectations and knowledge are integrated” (3, p.117). Knowledge of musical styles, music theory and standard performance practices are all contributing factors to fluent SR.

Undergraduate piano students typically recognise the importance of good SR skills for their careers, yet many report weakness in this area and a lack of SR training (4). Research suggests that pianists’ sight-reading fluency, as measured by the eye-hand span, is a unique characteristic of each player and is developed over a long period of time (5). Therefore, short-term interventions aimed at improving sight-reading are likely to have minimal impact on this skill. Mishra’s ( 6, 7, 8) meta-analyses of over 60 years of research into SR have identified effective strategies for improving SR as training in aural skills, solfége, composition, and improvisation. These approaches have been confirmed by Zhukov et al. (9) who implemented a hybrid approach to SR training that included rhythm training, familiarity with different musical styles, and ensemble work combined into a new curriculum for higher education. In a separate study the entrenched myth that informal practice improves sight-reading was also debunked (10).

Research into eye-movement (EM) during music SR is a niche area and has focused largely on eye-hand span and perceptual span of musicians (for reviews, see 11, 12). Most of the research on EM during music SR has investigated pianists, typically comparing piano sight-readers of different levels of expertise (e.g., 13, 14, 15, 16, 17, 18, 19, 20, 21). These studies show that expert piano sight-readers are able to see more notes ahead of their playing than do novices. For example, Waters, Underwood, and Findlay (21) report that expert pianists used fewer eye fixations, were quicker to notice differences/similarities between presented note patterns and able to process larger chunks of music than novices. Similarly, Truitt et al. (20) showed that expert piano sight-readers had shorter fixations and larger eye-hand spans than less skilled readers. In a more recent study Penttinen et al. (22) found that piano major students exhibited shorter fixations and larger eye-hand spans than less experienced music education majors. Huivinen et al. (23) proposed a new concept of eye-time span, showing that complexity of music affects the lengthening of looking ahead of playing and the lengthening of incoming saccades in university pianists.

All of these studies utilised a single line of music in their experiments because when pianists are reading two lines in treble and bass clefs simultaneously this results in a zigzag EM pattern different from other instrumentalists and singers who read a single line of music. However, pianists usually play with two hands reading two lines of music at once: reading a single score line is atypical and may jeopardise the natural EM strategies pianists tend to employ when performing real music. A recent study by D’Anselmo et al. (24) showed that playing with one hand and both hands does have an impact on the visual processing of pianists, with right-hand asymmetry present when playing with two hands and left-hand asymmetry when playing with one hand. While this finding may affect interpretation of findings in piano EM research, nonetheless single-line reading of pianists provides a comparison to music reading of other single-line musicians.

Research into SR of non-pianists and particularly into EM of non-pianists during SR is limited. Silent music reading, with technical challenges of playing an instrument removed, has been a recent focus of research. A study by Silva and Castro (25) into EM during silent music reading showed that expert musicians grasp similarities/ differences in rhythmic patterns faster than non-musicians. Similarly, Penttinen et al. ( 26) reported that greater musical experience results in shorter fixations and longer saccades when silently reading folk songs. Wurtz et al. (27) investigated SR of violinists, reporting that their prediction skills were linked to expertise, but stylistic features of the work (e.g., rhythm, melody) and speed of playing were also factors that impacted on fluent SR. Goolsby (28) compared EM of 12 expert undergraduate sight-singers to EM of 12 weakest sight-singers. He reported that expert sight-singers scanned the entire piece searching for information, backtracking to double check particular details (“regressing”), while novice readers only looked at the next note. Expert sight-readers fixations were shorter (*M* = 377.4 ms) than novices (*M* = 473.9 ms), and music complexity had an effect on fixations and saccades, with simple melodies producing fewer and shorter fixations and more complex melodies resulting in shortest saccades.

In woodwind playing an early study by Schmidt (29) investigating EM of six players during SR by comparing two players, an expert and a novice, on each of the three instruments: flute, clarinet and alto saxophone. While no differences between the instruments were found, SR expertise affected the number of regressions. The difficulty of SR exercise was also a factor in fixations and regressions. Another early study by Thompson (30) investigated SR in 30 flute players, reporting that SR expertise was highly correlated with eye-performance span and music recall, parallel to similar findings amongst the pianists. More recent investigation into predictors of SR expertise among high school wind players highlighted reading comprehension, rhythmic audiation, visual perception and spatial reasoning as factors contributing to expert SR (31).

Beyond technical skill on an instrument, task related cognitive strategies have been shown to impact on musicians’ sight-reading abilities. These include the types of cueing mechanism an experienced sight-reader will apply immediately before performing from sight, such as scanning the music to observe the hardest section to perform, checking the tempo, and making oneself aware of the key and time signatures, and other expression markings (32, 33). By the third year of playing an instrument young students showed an increase in the use of beneficial SR strategies such as identifying key signature, time signature and scanning music for obstacles. However, there appears to be a ceiling at an upper-intermediate-to-advanced level of playing, typically around the age of 15, when further enhancement of performance skills does not translate into improved SR (34).

## Aims

The review of literature on EM during music SR highlighted the lack of research into woodwind over the past 25 years and therefore, the need to evaluate the findings of early studies in the light of recent technological developments in eye-tracking equipment that does not require participants to hold their head against a fixed apparatus. The more advanced eye-tracking software samples EM at a faster rate than was previously available and therefore, can capture more accurate data on the number and duration of fixations. Most of the previous research had compared expert and novice sight-readers, resulting in lack of knowledge regarding the possible differences in eye movements between intermediate and advanced woodwind players and the impact of music complexity on eye movements. Since piano SR research represents the largest body of EM studies, it is also necessary to benchmark the findings against the existing piano EM literature.

Given that almost no research has been undertaken on the topic, this study used standard and well established EM and music testing procedures to examine the SR capability of undergraduate woodwind students of intermediate-to-advanced levels of expertise as they sight-read music of increasing difficulty. Our aim was to analyse:

1)
  how EM fixations and fixation durations of the participants changed as the music notation became more challenging, and
2)
  the relationship between EM strategies employed by the participants and their level of expertise.

## Method

### Participants

After obtaining ethical approval, a call for volunteer woodwind players was distributed at an Australian university. Six undergraduate students (three flute and three clarinet players) agreed to participate in the study. As this study was exploratory and not aimed at establishing larger population norms, extensive testing was conducted with a small number of participants sufficient for data analysis (e.g., 35, 36), and consistent with previous research investigating eye movement in music (e.g., 14). The students’ age ranged between 18 and 22 ( *M*=19.67) and years of playing their instrument 6 to 15 ( *M*=11.17). Their general level of performance skill, as measured by examinations passed, was between Grade 6 (intermediate) and Diploma (advanced) as defined by the Australian Music Examinations Board. Table 1 provides the demographics of the sample.

**Table 1:** Sample demographics.

ID	Age	Sex	Years of learning	Exam level
P1	22	F	11	Diploma 2 (=10)
P2	19	M	15	Diploma 1 (=9)
P3	19	F	13	8
P4	19	F	9	7
P5	18	F	6	6
P6	21	F	13	6
Mean	19.67		11.17	7.67

### Equipment

The eye-tracking experiments were conducted in a specially equipped laboratory on campus, utilizing the Tobii TX300 Eye Tracker connected to a PC with Tobii Studio Version 3.4.5 software. This eye-tracker was embedded in the computer screen, thus allowing for a certain freedom of the participants’ head movement. The eye-tracker sampled the eye movement at the rate of 300Hz. Polycom Speakerphone was used to record playing and synchronise audio with the eye-tracker.

### Procedure

The experiments followed a strict protocol for each participant that included laboratory orientation, signing consent forms, sitting in a comfortable chair approximately 60 cm in front of the computer monitor, warming up (playing a few random passages on the instrument), eye-tracker calibration, running the experiment, checking for data collection, and completing a short exit survey. Each session lasted approximately 45 min in total, including orientation, testing and exit survey.

### Stimuli materials

The experiment began with a general slide explaining the procedures displayed on the monitor, followed by a “Relax for 10 seconds” slide that alternated with the presentation of musical examples. Sight-reading examples were sourced from the *Watkins-Farnum Performance Scale* ( 37) that has been used by researchers and music educators as standard SR tests for band instruments for over 60 years. The musical examples are the same for different instruments but transposed into a key suitable for each instrument. The first 11 out of 14 examples were deemed suitable for the expertise level of this sample and were presented in order of increasing difficulty.

To import the scores into eye-tracker, the scores were re-formatted using Sibelius music notation software, matching exactly the number of lines and bars per line to the original. The PDFs of scores were imported individually, one per slide. Each musical example in the *Watkins-Farnum Performance Scale* ( 37) has a set metronome speed. Metronome click was played at the required tempo while the participants scanned each example and turned off as soon as they began playing. Participants were instructed to start playing when they were ready and if their preview appeared rather long they were encouraged to begin. On completion of each example, researcher initiated the “Relax for 10 seconds” slide that automatically moved to the musical example after 10 seconds. The experiment continued until each participant attempted all musical examples.

## Analyses

### Scoring of audio files

The audio recordings of playing were scored by two experienced music educators/ researchers independently, following instructions in the *Watkins-Farnum Performance Scale* ( 37). The scoring template gives a maxim possible score for each example, counts only one error per bar (pitch or rhythm), arriving at the score per example and total score per test. Should the number of mistakes be greater than the overall possible score, the examiner is instructed to stop the SR test. In this study, we decided to let students continue playing to the end of each example because we deemed that stopping playing might cause participants more distress than struggling to keep going. However, when errors outnumbered the possible score, the score given was 0 to comply with the intent of the original scoring system.

### Eye movement analyses

For each slide and for each participant, the eye-tracker provided a stream of vector data indicating the x and y position of the eye as a function of time (resolution of XHz or X/100). This data stream was further segmented into the 11 individual musical examples (isolated with a buffer of 100ms before and after the time stamps for each piece), and fixation data were extracted, particularly the number of fixations and the duration of fixation were considered. Proprietary software provided an indication of both the fixation number as well as its duration and these values were extracted for further data analysis. Fixation information provided a direct indication of the cognitive extent to which the participants were required to inspect and time to acquire information to perform the piece of music. Thus, fixation data might provide an indication of expertise as the expert might require fewer fixations and/or less time for information acquisition.

### Statistical analyses

Data were analysed using GraphPad Prism (version 8, La Jolla, CA). All eye movement data and subsequent means were confirmed to be normally distributed using the Shapiro-Wilk test (38). Accordingly, repeated measures one-way ANOVA were conducted to examine effect of music difficulty on the number of fixations and fixation durations separately. Correlations (Pearson’s *r*) were also performed to determine the relationship between the number of fixations and fixation duration, and SR accuracy and Exam level. These analyses were corrected using the Benjamini-Hochberg method to control for multiple comparisons and the false discovery rate. This method adjusts the alpha value for each comparison by first assigning ranks to the p-values associated with each correlation and then the Benjamini-Hochberg critical values are calculated by the formula (i/m)/Q in which I is the individual p-value’s rank, m is the total number of tests, and Q is the false discovery rate (0.05) (see 39). In addition, effect sizes were calculated to provide an indication of the standard size of the difference between the variables.

## Results

### SR accuracy

Scoring of audio files of each participant for pitch and rhythm accuracy as per the instructions in *Watkins-Farnum Performance Scale* ( 37) identified two outliers, Participant 1 and Participant 5. Participant 1 scored the highest overall mean of 109 out of a possible 113 points and participant 5 the lowest score of 74.5 (see Table 2). Intra-class correlation was performed using SPSS (two-way mixed model) to determine the absolute agreement between the two raters (40). A high degree of agreement was found between the two raters with an ICC (average measure) of .949 and 95% confidence interval from 0.526 to 0.993 (F[5,5]=31.763, *p*<0.001).

**Table 2:** Scoring of SR accuracy.

Participant ID	Judge 1	Judge 2	Mean
P1	109	109	109
P2	104	104	104
P3	89	78	83.5
P4	94	86	90
P5	75	74	74.5
P6	101	96	98.5

Maximum score is 113; higher score indicates greater SR accuracy.

### Eye movements

**Figure 1.**
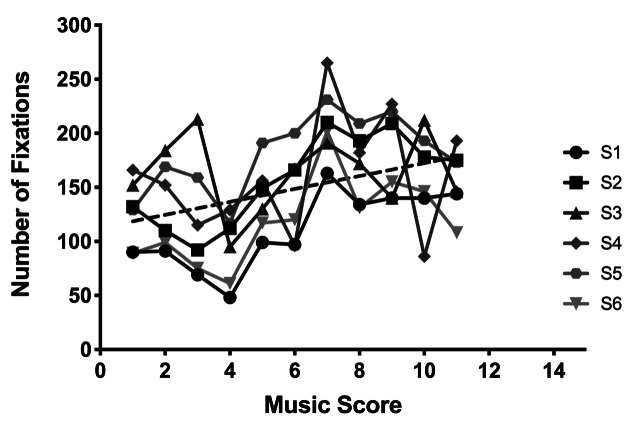
Number of fixations as a function of music score.

**Figure 2.**
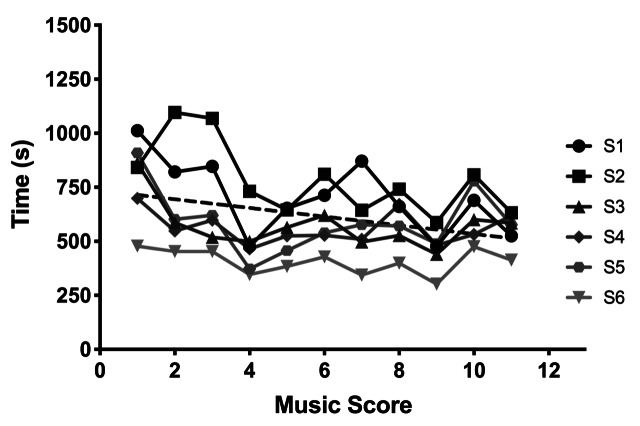
Fixation duration as a function of music score.

In Figures 1 and 2, the number of fixations and duration are shown as a function of the music score difficulty individually for the six participants (different grey circles), and mean and standard error of the mean values for number of fixations and fixation duration are reported in Table 3 for each music score. A Shapiro-Wilk test confirmed that the fixation and duration data (n=6) were normally distributed across all difficulty levels (*P*s>0.12). In regards to the number of fixations, a significant monotonic increase in the number of fixations was observed with increasing difficulty of the music (with means ranging from 57.8 to 79.3, F [10,50]=7.665, *p*<0.0001, η^2^=.605). At the same time fixation duration decreased significantly with the music score difficulty (with means ranging from 800.4 ms to 462.7 ms, F[10,50]=6.515, *p*<0.0001, η^2^=.867). Regression analysis confirmed these data trends which indicated that the slope of line for the best fit (5.98 for number of fixations and -20.06 for fixation duration as dash lines in Figures 1 and 2) significantly changes with the music score difficulty ( *P*s>0.0413).

**Table 3:** The number of mean fixation and fixation duration for each music score.

	Fixation	
Music Score	Number	Duration
One	126.33 (12.89)	864.92 (76.72)
Two	134.17 (16.02)	684.52 (96.05)
Three	120.50 (22.81)	684.33 (94.30)
Four	93.67 (13.27)	542.95 (55.74)
Five	140.33 (13.23)	538.03 (43.13)
Six	141.00 (17.50)	606.57 (56.44)
Seven	210.33 (14.26)	574.83 (72.19)
Eight	170.17 (12.93)	594.90 (49.98)
Nine	181.83 (16.79)	462.75 (37.87)
Ten	159.17 (18.43)	647.05 (54.70)
Eleven	156.50 (12.34)	558.52 (32.84)

Reported values are the mean (and standard error of the mean) for the 6 participants

These results show that as music score became more difficult, the number of fixations increased, but the duration of each fixation decreased.

**Figure 3.**
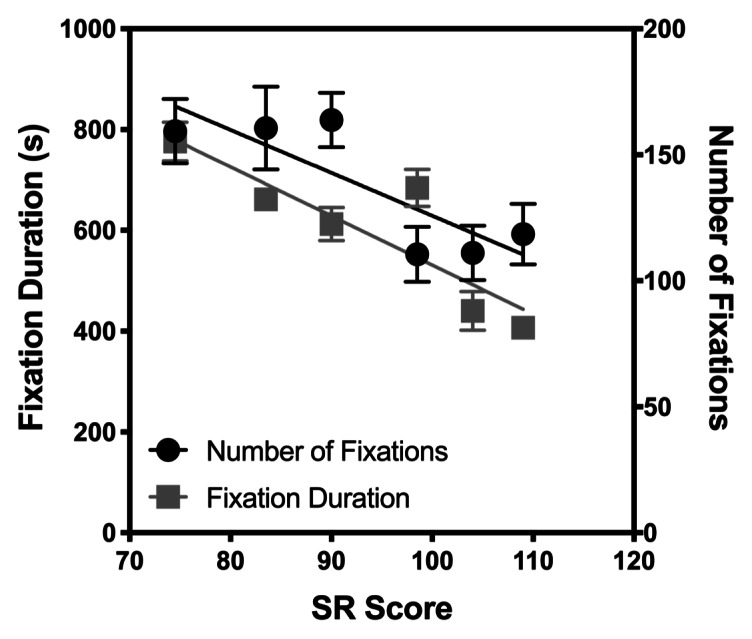
Eye movement versus SR accuracy.

**Figure 4.**
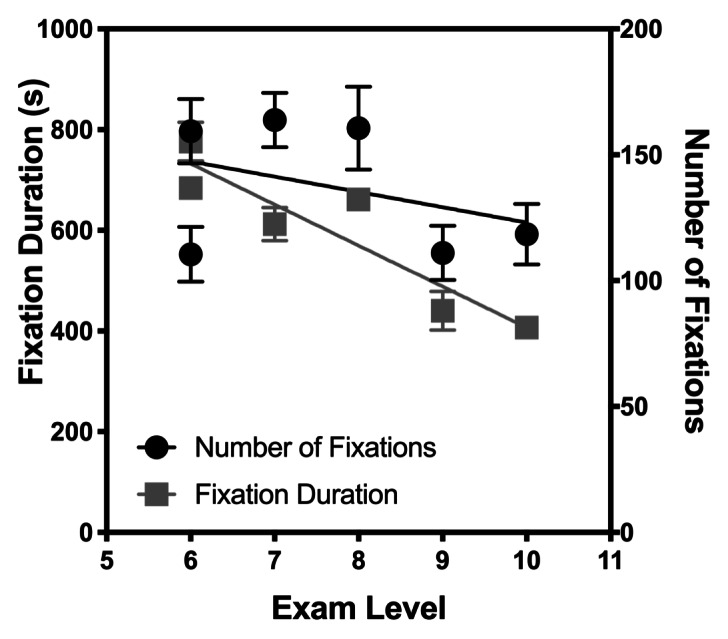
Eye movement versus level of exam passed.

In Figures 3 and 4, the number of fixations and fixation duration (different symbols) are plotted against the SR accuracy and Exam level respectively to investigate how they correlate. For fixation duration there is a significant negative linear trend, such that fixation duration reduces with SR score (with mean duration ranging from 776.3 ms (Score 1) to 406.8 (Score 11), Pearson *r*=-0.876, *p*=0.0221, Benjamini-Hochberg critical value of 0.025) and Exam score (Pearson *r*=-0.927, *p*=0.0222, Benjamini-Hochberg critical value of 0.05). Additionally, for each comparison, the slope of the best fitting line relating fixation duration with SR and Exam score was significantly different from zero, with Ps<0.0001. These results show that more experienced players with better SR skills required less time to process musical notation. However, there was only a significant change in the number of fixations when comparisons were made with the SR score (with means ranging from 110.5 to 163.8, Pearson *r*=-0.840, *p*=0.0363, Benjamini-Hochberg critical value of 0.05). The slope of the line of best fit was significantly different from zero, p=0.002. Thus, participants with higher SR skills utilised fewer fixations to acquire information visually.

## Discussion

This exploratory study investigated woodwind SR using modern eye-tracking equipment and focusing on the impact of task complexity and player expertise at intermediate-to-advanced level on EM strategies.

The findings show that participants’ EM did change as musical materials became more complex and challenging. The major finding of the study is that the number of fixations had increased and the duration of these fixations decreased significantly. This finding suggests that as the music became more complex, the musicians were challenged in a way that required them to process more information. Hence, the number of fixation increased. Interestingly, the durations of the fixations decreased significantly whilst they were doing this. This finding suggests that with increasing task difficulty participants are moving their eyes and saccading more often, but increasing their effort (perhaps through more focussed attention), as indicated by a reduction in fixation duration. The number of fixations and fixation duration was unique to each of these woodwind performers, and it is likely that *this had been developed over many years of musical training*. Fixation duration may also indicate a limit of cognitive processing speed that is specific to each person and is a result of convergence of many educational and personal factors. Only three studies into EM during SR have documented the impact of task complexity on EM, reporting significant effect on fixations (23, 28, 29). The lack of research in this aspect of EM during SR highlights the need to consider task difficulty in analyses of EM data as a factor that might impact EM strategies.

Previous research has largely focused on comparing EM of expert and novice sight-readers, reporting that experts tend to use more effective EM strategies such as looking ahead, processing larger chunks of information, scanning for difficulties and having a larger eye-performance span (20, 21). Our study investigated EM during SR by *intermediate* and *advanced* higher education students. The results show that fixation duration was correlated with SR accuracy score and examination level, with better sight-readers (higher SR scores) and more experienced players (higher level of examination passed) demonstrating fewer fixations and shorter fixation durations than the weaker sight-readers and less experienced students, suggesting that experts were able to process the information quicker. Shorter fixations by expert sight-readers were previously reported in experiments involving instruments other than woodwind (20, 22, 26, 28). Fewer fixations by experts have not been previously reported in the literature and this result will need to be replicated in future studies with larger samples of participants.

Our findings suggest that EM strategies exhibited by undergraduate woodwind players support the trends previously reported in SR research.

## Conclusions and Implications

This pilot study has shown that expertise is a factor in EM during woodwind SR, with more experienced players and better sight-readers demonstrating different EM from less experienced and weaker sight-readers. Complexity of music task was also a factor that should be investigated further.

Future research needs to validate our results in larger samples of woodwind players of various levels of expertise: beginners, intermediate and advanced students. This will enable music educators to develop better teaching strategies that will target weaknesses in music reading at crucial skill development windows.

Technological advances in recent years have made it possible to carry out EM research in the field instead of laboratories, with researchers only needing a laptop, a thin strip eye-tracker and relevant software. These advances open up the area for more fascinating projects on EM in music performance and other areas requiring processing of visual information.

## Ethics and Conflict of Interest

The authors declare that the contents of the article are in agreement with the ethics described in http://biblio.unibe.ch/portale/elibrary/BOP/jemr/ethics.html and that there is no conflict of interest regarding the publication of this paper.
